# South West Orthopaedic Club

**Published:** 1969-07

**Authors:** 


### SOUTH WEST ORTHOPAEDIC CLUB

A meeting of the South West Orthopaedic Club was held at the Musgrove Park Hospital, Taunton, on Saturday, 16th November, 1968, under the Chairmanship of Mr. R. F. Winckworth.

The morning session consisted of a large clinical demonstration followed by a discussion of the problems which emerged from it.

During the afternoon the following papers were read :—

*Occipito-Cervical Fusion.*

Mr. H. J. Hambury (Swansea) described two patients who had undergone this operation. The first had a pathological dislocation of the atlanto-axial joint due to osteomyelitis. The second showed congenital forward subluxation. Other indications for the operation were described, namely rheumatoid arthritis, injury, and severe muscular paralysis. Mr. Hambury offered a contribution to the operative technique : two drill holes were made transversely in the substance of the occipital bone so that wires could be passed through them.

*Segmental Fractures of the Tibia.*

Mr. J. R. Clough (Bristol), reported on 47 patients with double fractures of the shafts of the tibia. Union was delayed beyond five months in two-third, and bone grafting procedures were needed in one quarter of these patients. Neither the upper nor the lower fractures had a greater propensity to develop delayed union. The shorter the intermediate fragment the more likely was delayed union in the associated fractures, the incidence being 40 per cent in those patients with a fragment of at least half the length of the shaft, rising to 80 per cent when the fragment length dropped to a quarter or less of the shaft length.

A quarter of the patients had internal fixation of their fractures. Figures were too small to allow firm conclusions to be drawn but it was shown that in these patients union was no slower than in the conservatively treated group and there had been no serious complications. The risk of further impairment of the blood supply to the middle segment was felt to be a less important argument against internal fixation than the risk of provoking necrosis of the skin overlying the fractures, i.e. skin already damaged in these direct trauma injuries.

*Fractured Head of Radius in Adults.*

Mr. I. M. Pinder (Bristol). This study consisted of a retrospective series of over 200 cases and a prospective series of over 100 cases. These cases were classified using Mason's classification into 3 types based on the radiological appearance.

The main problem encountered was a failure to regain full extension of the elbow after a fractured head of radius. This failure was felt to be due to a mechanical block, not demonstrable radiologically, the probable cause of which was fibrosis of the anterior capsule of the elbow joint, due it was felt to an organised haemarthrosis. Hence, aspiration of the elbow was undertaken, using a lateral approach, alternate cases of Type 1 (56 cases) average aspirate 5 mls., and Type 2 (41 cases) average aspirate 7.5 mls. In Group 1 all cases were immobilised in a collar and cuff in full flexion for 3 weeks. In Group 2 treatment was dictated by the range of movement regained after aspiration. If over two-thirds, treatment was as for Group 1, but if the range was less, excision of the head of the radius was performed. In Group 3 excision of the radial head was routine.

*Results*:

1. Complete relief of pain in all aspirated cases. Aspiration enabled an accurate forecast to be made of the range of movements, the ultimate range of which was almost always greater. It enabled a more complete recovery to be made in Type 2 fractures (58 per cent full movements compared with 35 per cent complete recovery in the control series at 6 months).
2. Damage to the medial ligament is a common finding and means a slowed and less complete recovery; this is confirmed radiologically by a bony spur becoming apparent some weeks later.
3. A full range of pronation/supination was almost invariable in all groups.
4. Just over 50 per cent of the fractures resulted from direct injury, a fall on to the hand.
5. 65 per cent of the patients were women.

**Mr. H. K. Lucas (Bristol)** gave an interesting account of a patient who is now aged 60, who was operated on at the age of 10 by Mr. Hey Groves. The original paper was given in a Hunterian Lecture 10 years later and the actual lecture note was quoted. It appeared to be a case of fibrous dysplasia of the humerus and was treated by resection and replacement with an intra-medullary beef bone peg fixed with rivets. The patient later won the weight putting at Cambridge University and is still free of disability.

*Symposium on the Management of Bony Metastases.*

The following papers were given :—

**Dr. R. C. Tudway (Bristol)** on the place of radiotherapy and cytotoxic drugs. It was stressed that there is no growth so sensitive that both it and its metastases can be totally destroyed by the amount of radiotherapy and cytotoxic drugs that the patient as a whole can tolerate.

**Mr. R. D. Rowlands (Taunton)** discussed the indications for oophorectomy and adrenalectomy for carcinoma of the breast with metastases. He favoured oophorectomy for patients in the younger age groups who were hormone sensitive. He did not consider that the operation had any value as a prophylactic procedure. Adrenalectomy was thought to hold out only minor advantages over oophorectomy and is a bigger operation.

**Mr. P. H. Huggill (Taunton),** described his experience with hypophysectomy for carcinoma of the breast and prostate with metastases. His review was based on a personal experience of 65 patients. He considered that the operation gave rapid relief from bone pain and some regression of the metastases. He did not expect a cure but often achieved 2 or 3 years of enjoyable survival. He considered that the operation should be performed as soon as there was evidence of further metastatic spread, for example after oophorectomy.

**Mr. A. E. Jowett (Taunton)** described the indications for internal fixation for pathological fractures. The various methods used were described and there was some discussion on the wisdom of prophylactic nailing in selected cases.

